# Retropharyngeal abscess-like as an atypical presentation of Kawasaki disease: a case report and literature review

**DOI:** 10.1186/s12969-023-00812-z

**Published:** 2023-04-13

**Authors:** Rim Kasem Ali Sliman, Joris M. van Montfrans, Najwan Nassrallah, Mohamad Hamad Saied

**Affiliations:** 1 Pediatric Department, Carmel Medical Center, Michal 7 St., 3436212 Haifa, Israel; 2 Technion Faculty of Medicine, Haifa, Israel; 3 Department of Pediatric Immunology and Infectious Diseases, Wilhelmina Children’s Hospital/University Medical Center, Utrecht, The Netherlands; 4 Department of Radiology, Carmel Medical Center, Michal 7 St., 3436212 Haifa, Israel

**Keywords:** Kawasaki disease, Retropharyngeal abscess, Retropharyngeal collection, Antibiotics unresponsive collection, Inflammatory disease

## Abstract

**Background:**

Kawasaki disease (KD) is a systemic inflammatory condition primarily affecting young children. Although 90% of KD patients present with variable head and neck manifestations, especially cervical lymphadenopathy, peritonsillar, retropharyngeal and parapharyngeal involvement are uncommonly reported as initial manifestations of KD.

**Case report:**

Eight-year-old girl with prolonged fever, clinical and a radiological picture suggestive of retropharyngeal abscess, unresponsive to three changes in the antibiotic regimen and surgical drainage. The disease progressed with the development of additional signs and symptoms as non-purulent conjunctivitis (with uveitis), mucosal involvement (strawberry tongue and cracked lips), edema of her hands and feet, and arthritis. A diagnosis of Kawasaki disease was reached with complete remission after Intravenous Immunoglobulin (IVIG) treatment.

In addition, we present a literature review of similar cases reported in the last thirty years.

**Conclusion:**

Kawasaki disease requires a high index of suspicion and awareness of unusual presentations. It should be kept in mind as one of the differential diagnosis of patients with febrile inflammation of the retropharyngeal and parapharyngeal spaces who do not respond to antibiotic treatment in the relevant clinical context.

## Background

Kawasaki disease (KD) is an acute, self-limiting vasculitis of childhood, typically presenting before the age of five years, with a peak age incidence between 6 months and 2 years of age [1–6]. The vasculitis predominantly affects medium-sized arteries, with a striking predilection for the coronary arteries, leading to coronary artery aneurysms (CAAs) in 15- 25% of untreated cases, and thus causing the most important life-threatening complication of KD [1–4]. KD is the major cause of acquired heart disease in the developed world, with consequences including myocardial infarction, ischemic heart disease, and sudden death [1, 3, 5, 6].

Due to the non-specific symptoms and a lack of specific confirmatory laboratory tests, the diagnosis is based on clinical criteria. This is often challenging as only 40% of KD patients present with conclusive clinical criteria, the remainder presenting with incomplete or atypical symptoms [1, 6, 7].

The incidence of CAA decreases to < 5% after timely treatment with high-dose intravenous immunoglobulin (2 g/kg/day) therapy, which is most effective if commenced within the first ten days of signs. Awareness of unusual clinical KD manifestations is important, as it may raise the index of suspicion and expedite treatment [5, 8, 9].

In this article, we describe a patient who presented with an atypical retropharyngeal abscess-like lesion, unresponsive to several antibiotics regimens, as well as surgical drainage, but promptly responded to immunoglobulin treatment once a diagnosis of KD was suspected. In addition, we present a literature review of similar cases spanning the last thirty years.

## Materials and methods

A literature search was conducted, and the primary database was PubMed (Medline), Embase, and Google Scholar.

The keywords were Kawasaki disease, Pediatrics, and Retropharyngeal abscess. Fifteen articles were found, with sixteen case reports describing pediatric patients presenting with retropharyngeal abscess, later diagnosed with Kawasaki disease (by fulfilling the criteria) or atypical Kawasaki (by fulfilling part of the criteria, as well as a significant and fast clinical improvement after IVIG treatment (Table 1).

**Table 1:** Summary of the characteristics of the case reports in the literature review

	**Source**	**Age (years)**	**Sex**	**Fever (Days)**	**Initial additional presentation**	**Antibiotics**	**Radiological findings**	**Surgical findings**	**Echocardiography**
1	Pontell 1994 [10]	5	M	1	Left neck pain, stiffness	Yes	A 3-cm hypodense retropharyngeal lesion without peripheral enhancement	Normal pharynx	Small pericardial effusion
2	Park 1997 [3]	4.5	M	7	Acute otitis media, torticollis, neck mass	Yes	Left retropharyngeal mass extending laterally into the neck; several small areas of hypolucency within the mass and associated ring enhancement	Not done	Normal
3	McLaughlin 1998 [11]	4	M	2	Neck swelling, torticollis	Yes	Extensive retropharyngeal edema crossing the midline and extending from the level of nasopharynx inferior to thoracic inlet	Not done	Normal
4	Rook 1999 [12]	4	M		sore throat, drooling	yes	Retropharyngeal abscess	Not done	Normal
5	Homicz 2000 [13]	6	F	2	Anterior neck pain, torticollis, odynophagia	Yes	A 2.8-cm low-density mass without enhancement in the retropharyngeal space	No abscess found	Normal
6	Gross 2001 [14]	9	M	7	Torticollis, headache	Yes	Retropharyngeal soft tissue swelling without enhancement	No fluid collection	Normal
7	Miao-Chiu Hung 2006 [9]	2.4	M	2	Irritability, neck swelling	Yes	Ill-defined low-density lesions in bilateral aspects of the retropharyngeal space around the level of the oropharynx	Not done	Normal
8	Langley 2008 [15]	3	M	3	Right sided neck swelling	Yes	Edema and inflammatory changes in the retropharyngeal space with associated right cervical adenitis	Not done	A small left coronary artery aneurysm
9	Ganesh 2008 [16]	8	M	1	Left neck pain, torticollis, trismus	Yes	Ill-defined hypodense lesion extending from the C2-C6 vertebral level in the posterior pharyngeal space with no contrast enhancement	2 ml pus, sterile cultures	Fusiform aneurysm of the proximal and mid right coronary artery 6.5 × 6 mm
10	Choi,2010 [2]	3	M	5	Bilateral conjunctival injection, left neck pain	Yes	Low density lesion with an irregular thick wall in the left lateral node, suggesting an abscess, and multiple lymph node enlargements in the left posterior cervical space	Purulent fluid collected	Initial- perivascular brightness around both coronary arteries Repeated—showed mild dilation of the left main coronary artery (2.5 mm)
11	MacHaira 2012 [4]	1.3	M	1	odynophagia, torticollis, irritability	Yes	A hypodense lesion extending from C2 to C6 vertebral level in the posterior pharyngeal space and narrowing of the upper respiratory tract	Not done	Normal
12	Kritsaneepaiboon 2012; [7]	10	F	5	Irritability, Neck swelling	Yes	Bilateral cervical lymphadenopathies and retropharyngeal low-attenuation area with a mildly enhancing rim which extended to the bilateral posterior cervical spaces and downward to the thoracic inlet level	0.5 ml of serosanguinous fluid	Perivascular brightening and cuffing of right coronary artery and inner wall irregularity of left anterior descending artery
13	Kritsaneepaiboon 2012; [7]	11	M	4	torticollis	Not documented	A low-attenuation area without contrast rim enhancement at the retropharyngeal space with extension into bilateral parapharyngeal spaces and posterior cervical spaces down to the level of C5	Not done	Pericardial effusion
14	Aldemir-Kocabaş 2014 [8]	9	M	7	Sore throat, left-sided neck swelling	Yes	Prevertebral hypodense soft tissue compatible with abscess formation, extending from C2 to C5 cervical vertebrae, and deep cervical–retropharyngeal necrotic lymphadenopathy left to midline	Not done	Normal
15	Connell 2018; [3]	14	M		Odynophagia, drooling, neck swelling	Yes	A 31 × 24 mm soft tissue mass arising from the left palatine tonsil fossa with an area of central low attenuation. There was a large 3 × 3 cm lymph node anterior to the sternocleidomastoid muscle	Not done	Initial- prominent and irregular left main coronary artery The RCA was diffusely dilated and irregular, up to 5.4 mm Repeated- persistent diffusely dilated RCA up to 5 mm
16	Chiara 2019 [1]	4	M	3	Neck pain with right cervical swelling	Yes	Multiple right side lymph nodes with a tendency towards confluence and an infiltration with intense enhancement of the sternocleidomastoid, parapharyngeal and retropharyngeal tissues with preserved respiratory space	Not done	Normal
17	Present 2019	8	F	2	Pain, swelling and redness at left cervical area	Yes	Swelling at the left retropharyngeal region with a hypodense lesion up to 45 mm with multiple enlarged and inflamed lymph nodes	A little serous fluid	Normal

Kawasaki disease criteria: Fever persisting at least five days with 2 At least four of the five principal clinical features: i) Changes in extremities Acute: Erythema of palms, soles; edema of hands, feet Subacute: Periungual peeling of fingers and toes in weeks 2 and 3 ii) Polymorphous exanthema (diffuse maculopapular, urticarial, erythroderma, erythema-multiforme like, not vesicular or bullous) iii) Bilateral bulbar conjunctival injection without exudates iv) Changes in lips and oral cavity: erythema, lips cracking, strawberry tongue, diffuse injection of oral and pharyngeal mucosae v) Cervical lymphadenopathy (> 1.5 cm diameter), usually unilateral [17].

## Case report

An eight-year-old girl, previously healthy, presented to the emergency department with three days of fever associated with left-side swelling of the neck, tenderness, and torticollis with no improvement after 24 h of oral antibiotic treatment. The physical examination revealed bilateral cervical lymph node enlargement. Her positive laboratory findings included leukocytosis of 16,800/μl with 85% neutrophils, C-reactive protein of 13 mg/dL (normal range 0–0.5 mg/dL), and a throat swab culture that showed no growth. EBV and CMV serology were negative. An ultrasound showed multiple enlarged lymph nodes, the largest with a 25 mm circumference. She was admitted, and IV Amoxicillin\Clavulanic Acid was commenced for suspected cervical lymphadenitis.

**Fig. 1:**
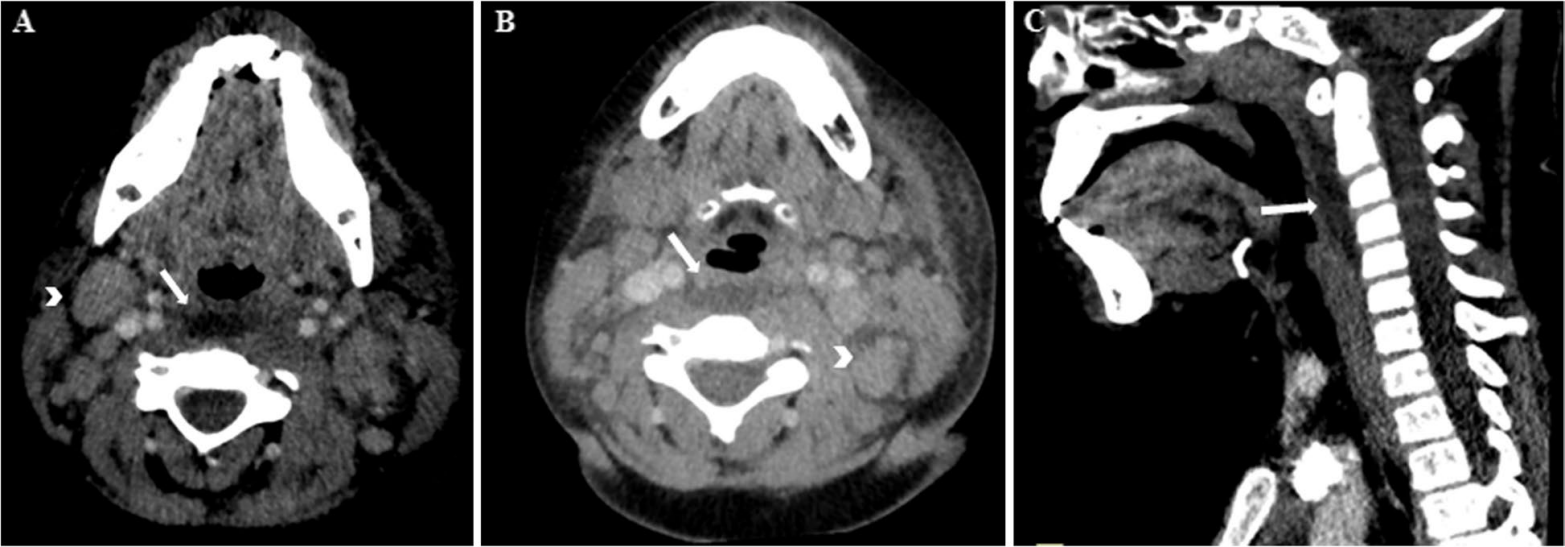
**A** and **B** Axial enhanced CT images demonstrate collection (arrows, **A** and **B**) filling from side to side in retropharyngeal space, with bilateral lymphadenopathy (arrowheads **A** and **B**). **C** Sagittal enhanced reformatted CT image shows fluid collection (arrow) expanding in retropharyngeal space

Despite antibiotic treatment, her fever continued, as well as the neck swelling. A cervical CT was performed on the 4^th^ day of fever, demonstrating left retropharyngeal swelling with a hypodense lesion of 45 mm with a suspected abscess (Fig. 1A-C), as well as multiple enlarged and inflamed lymph nodes. Expansion of antibiotic coverage to ampicillin sulbactam also showed no clinical improvement. Subsequently, she underwent surgical drainage, which showed minimal serous fluid and sterile bacterial cultures. A third antibiotic change to Ertapenem, was also without any clinical improvement. On the 8^th^ day of fever, symmetrical arthritis of the PIPs, MCPs, and MTPs joints appeared. On the 9^th^ day of fever, non-purulent conjunctivitis, as well as a strawberry tongue with swollen dry lips were noted. An ophthalmological examination showed mild anterior uveitis. Echocardiography was normal.

At this point, she was diagnosed with Kawasaki disease, and the administration of a single dose of IVIG (2 Gram\kg) led to a rapid clinical improvement and resolution of fever and other signs and symptoms. At her follow-up visit two weeks later, she displayed a desquamation of the skin of the tips of the thumb and third finger. At the follow-up four weeks later, the echocardiography of the coronary arteries was normal.

## Discussion

More than 50% of KD patients have atypical presentations that may result in delayed or missed diagnosis and, consequently, high rates of vascular damage. We present in this paper case of KD that presented with a retropharyngeal abscess-like lesion, which is a rare presentation.

Previous literature has shown that 90% of KD patients present with variable head and neck manifestations such as facial exanthema, conjunctivitis, oral mucosal changes, and pharyngitis. Less common presentations are otitis media, torticollis, deep neck infection-like symptoms, meningismus, and acute tonsillitis have been described [7].

Lymphadenopathy as a KD criterion is the least common diagnostic feature (50–75%), as the incidence of the other criteria occurs in 80–90% of the cases. Lymphadenopathy is described as the initial presenting symptom in 12%. Deep neck infection-like presentation is described in only less than 5% of all the patients with head and neck manifestations [2, 9, 13]. Signs and symptoms of retropharyngeal involvement, such as stridor, neck pain, and dysphagia, are less common in KD than in retropharyngeal abscesses [7].

Peritonsillar, retropharyngeal and parapharyngeal swelling are uncommonly reported as initial manifestations of KD. The precise pathophysiology of the association of KD with retroparapharyngeal pathology is unclear, with inflammation and edema hypothesized as the main mechanisms [3, 8].

Tashiro et al. described the typical ultrasound appearance of lymphadenopathy in KD patients as multiple hypoechoic–enlarged nodes forming a palpable mass resembling a cluster of grapes. This is in comparison to the appearance of bacterial lymphadenitis, described as a well-defined mass with a large central hypoechoic area surrounded by satellite normal-sized lymph nodes [18].

Pontell et al. reported the first case of KD mimicking a retropharyngeal abscess in 1994 [9, 10]. Glasier et al. reported that there might be an overlapping CT density number between cellulitis, adenopathy, and abscess, which may lead to false interpretations [19].

Poor response to antibiotic therapy and a negative aspirate culture may be additional diagnostic clues to correctly diagnosing KD [2].

Uveitis can be part of KD during the first week of illness; about three-fourths of children are photophobic, a consequence of anterior uveitis, 64 which peaks between 5 and 8 days of illness and is more common in children over 2 years old [20].

Our literature review found sixteen similar reported cases in the last thirty years. We summarized the clinical presentation, imaging findings, surgical and echocardiographic findings, and antibiotics treatment in Table 1.

The KD patients with abscess-like lesions were predominantly males (82%), with an age range from 10 months to 9 years, with a mean age of 5 years, which is considered higher than the average age of patients with KD. The majority of the cases (94%) initially presented with fever and neck swelling, without additional clinical criteria, leukocytosis. Leukocytosis was in all patients with an average of 18,500 WBC/μL. All the cases were initially considered for possible deep-neck bacterial infection, prompting antibiotic therapy and imaging studies. CT\MRI imaging was performed in all the cases, with findings suspicious for retropharyngeal disease, but in the majority, without enhancement. Indeed, low-density cervical lesions demonstrating minimal to no enhancement should raise the possibility of KD.

Intravenous antibiotics were administered in 16 patients (94%), and surgical drainage was attempted in 7 patients (41%), with two 2 cases (11%) of purulent fluid, only one of them being culture positive for Staphylococcus aureus.

As with our patient, the diagnosis of KD was made in these cases when the fever persisted and other characteristic features developed. The diagnosis was delayed beyond nine febrile days in 5 patients (30%). All the patients had a dramatic and fast clinical improvement with resolution of fever within 48 h after IVIG administration; the majority even showed improvement in the first 24 h. Most of the patients responded to one dose of IVIG; in only two cases, two doses of IVIG were administered due to slow response, and in these two cases, the diagnosis was not delayed and, they were diagnosed on the 7^th^ day of fever.

41% of the patients had cardiac manifestations compared to less than 20% in the general pediatrics pediatric population who are diagnosed with Kawasaki disease.

In summary, we present our patient and review 17 cases reported over 30 years (since 1990), presenting with clinical presentation and radiological findings consistent with the presence of a retropharyngeal abscess. Nearly all patients were treated with antibiotics and surgical exploration without improvement. All the cases responded promptly to IVIG therapy. Cardiac involvement was more common than expected in other cases of KD.

## Conclusion

Kawasaki disease requires a high index of suspicion and awareness of unusual presentations. In our case and literature review, Kawasaki disease mimicked a retropharyngeal abscess that was refractory to antibiotics and surgical intervention.

Thus, Kawasaki disease should be kept in mind as one of the differential diagnosis of patients with febrile lymphadenitis and/or retropharyngeal abscess who do not respond to antibiotic treatment in the relevant clinical context. This can prevent delay in diagnosis and detrimental sequelae.

## Data Availability

Raw data for this study (i.e., specific data extracted from each publication are available upon request).

## Abbreviations

CAA: Coronary artery aneurysm
CT: Computerized Tomography
IVIG: Intravenous Immune Globulin
KD: Kawasaki disease
MCP: Metacarpophalangeal
MTP: Metatarsophalangeal
PIP: Proximal interphalangeal
